# Emerging Roles of B56 Phosphorylation and Binding Motif in PP2A-B56 Holoenzyme Biological Function

**DOI:** 10.3390/ijms25063185

**Published:** 2024-03-10

**Authors:** Yanqiao Zhang, Haonan Jiang, Haimeng Yin, Xinyuan Zhao, Yali Zhang

**Affiliations:** 1 School of Medicine, Nantong University, Nantong 226001, China; zhangyanqiao0202@126.com (Y.Z.); 15851293399@163.com (H.J.); yinhaimeng27@163.com (H.Y.); 2 Department of Occupational Medicine and Environmental Toxicology, School of Public Health, Nantong University, Nantong 226019, China

**Keywords:** PP2A-B56 holoenzyme, phosphorylation, LxxIxE motif, substrate specificity, molecular mechanism

## Abstract

Protein serine/threonine phosphatase 2A (PP2A) regulates diverse cellular processes via the formation of ~100 heterotrimeric holoenzymes. However, a scarcity of knowledge on substrate recognition by various PP2A holoenzymes has greatly prevented the deciphering of PP2A function in phosphorylation-mediated signaling in eukaryotes. The review summarized the contribution of B56 phosphorylation to PP2A-B56 function and proposed strategies for intervening B56 phosphorylation to treat diseases associated with PP2A-B56 dysfunction; it especially analyzed recent advancements in LxxIxEx B56-binding motifs that provide the molecular details of PP2A-B56 binding specificity and, on this basis, explored the emerging role of PP2A-B56 in the mitosis process, virus attack, and cancer development through LxxIxE motif-mediated PP2A-B56 targeting. This review provides theoretical support for discriminatingly targeting specific PP2A holoenzymes to guide PP2A activity against specific pathogenic drivers.

## 1. Introduction

Dynamic protein phosphorylation is a post-translational modification that plays an important role in regulating apoptosis, proliferation, signaling, the cell cycle, and various other cellular processes that maintain normal homeostasis. Protein phosphorylation is a highly functionally coordinated and temporally regulated process by protein kinases and phosphatases. Disorders of this balance underlie the pathogenesis of many human diseases, such as cancer [1]. While there are approximately 500 identified protein kinases, paradoxically, there are only about 60 identified phosphatases [2]. This imbalance led to the early misperception that phosphatases have relatively nonspecific enzymatic activities, however, many protein phosphatases are composed of multimeric protein complexes, allowing for the assembly of a repertoire of structurally distinct holoenzymes, serine/threonine protein phosphatase 2A (PP2A) is a perfect example. The intricate function of PP2A is supported by its complex structure, in which a structural subunit A forms a core dimer with the catalytic subunit C and then polymerizes into heterotrimeric phosphatases with one of the regulatory B subunits from four families (B/B55, B′/B56, B″/PR130/70/72, and B‴/STRN), many of which are further diversified by alternative splicing. Of note, these regulatory B subunits determine the substrate specificity, localization, and consequent physiologic functions of different PP2A trimers [3,4,5,6]. Due to its crucial role in maintaining cellular homeostasis, the biogenesis of PP2A is tightly regulated at various levels, beginning with the presence of unbound PP2A C [7,8,9]. The binding of free PP2A C to alpha 4 (α4) results in the inactivation of the PP2A C catalytic site and protection from degradation, thereby preventing unregulated catalytic activity. This process maintains a sufficient pool of PP2A C that can be reactivated by the PP2A phosphatase activator (PTPA) to quickly respond to changes in cellular homeostasis [8,10]. It has been demonstrated that methylation of the C-subunit (Leu309) by LCMT-1 facilitates the binding of methylation-sensitive B-subunits, such as B55α, B56α, B56β, and B56ε. Methylation is reversed by PME-1, allowing B-subunits like those from the STRN family to bind to the C-subunit. In addition to inhibiting the interaction of PP2A-C with PTPA, phosphorylation at Thr304 of the C-terminal tail is crucial for directing B-subunit binding and regulating the enzymatic activity of PP2A [11]. Phosphorylation of Tyr307 at the C subunit inhibits the PP2A holoenzyme assembly effectively, however, the functional implications of Tyr307 phosphorylation remain unclear due to the lack of specific antibodies [12].

The B-type subunits confer distinct functions and characteristics of the trimeric PP2A complex. Among the various regulatory B subunits, the B56 family is the largest of the four regulatory subunit families, comprising at least seven different members (α, β, γ1, γ2, γ3, δ, and ε). Overall, PP2A-B56 has been established to function as a tumor suppressor, diminishing the activation of master oncogenic regulators, including extracellular signal-regulated kinases (ERK), protein kinase B (AKT), and p53. In response to various growth factors, the phosphorylation of B56 by ERK allows IEX-1 to increase PI3K/AKT activity by preventing AKT dephosphorylation on residues Thr308 and Ser473. It has been shown that dephosphorylation of B56γ3 at Ser510 strengthens the tumor-suppressive function of P53 by transcriptional activation [13].

It is worth mentioning that all the B56 family members except B56γ1 are phosphoproteins, which means B56 itself can be regulated by reversible phosphorylation and dephosphorylation [14]. In fact, the regulation of B56 phosphorylation is one mechanism by which PP2A holoenzyme function can be regulated in terms of location, binding to substrate, and phosphatase activity. The fact extends the potential (patho)physiological roles of PP2A-B56 [13,15,16,17,18,19]. More importantly, the discovery of the B56 docking motif-LxxIxE in recent years increased our understanding of how PP2A-B56 recognize their substrates and guided further investigations of diverse signaling circuits formed by PP2A-B56 holoenzymes in various cellular processes [20,21,22]. As a consequence, competitive inhibitors with excellent potency and specificity toward PP2A-B56 and phosphorylation sites targeted by PP2A-B56 holoenzymes have been developed and identified [3,21]. Overall, the properties of the B56 subunit phosphoprotein and the identification of its specific short linear motif (SLiM) make PP2A-B56 a multirole protein phosphatase in regulating cellular processes.

Based on the discovery of B56 phosphorylation’s ability to regulate PP2A-B56 function and its specific docking motif, this review will cover the functions of PP2A-B56 and their contribution to deciphering the mechanisms of PP2A substrate recognition and lay the foundation for selectively targeting B56 family subunits to regulate B56-specific PP2A holoenzyme activity and specific phosphoprotein targets. This review contributes to shifting the treatment of diseases associated with PP2A dysfunction from indiscriminately targeting PP2A holoenzymes to selectively targeting specific PP2A holoenzymes to guide PP2A activity against unique pathogenic drivers.

## 2. The Important Role of B56 Phosphorylation in PP2A-B56 Function and Its Mechanism

### 2.1. Biological Function of B56 Phosphorylation

**Figure 1:**
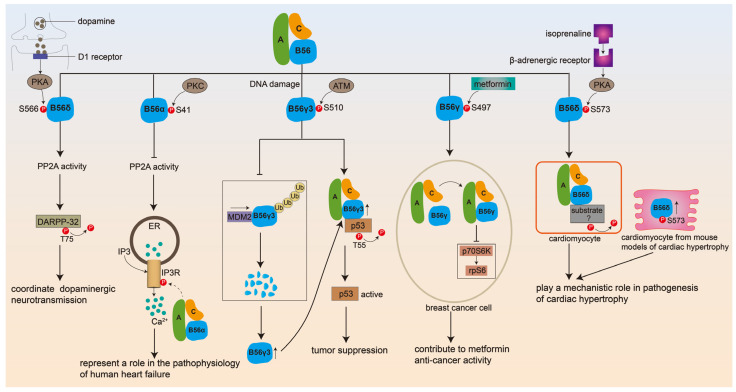
Effect of B56 phosphorylation on PP2A-B56 holoenzyme function. Dopamine D1 receptor-mediated phosphorylation of B56δ at S566 by PKA increases PP2A activity and the resulting dephosphorylation of DARPP-32 coordinates the efficacy of dopaminergic neurotransmission in striatal neurons; the phosphorylation of B56α at S41 by PKC inhibits PP2A activity and thus modulates IP3R activity, leading to an increase in the IP3-mediated Ca^2+^ release from endoplasmic reticulum (ER); In response to DNA damage, B56γ3 phosphorylation at S510 by ATM disrupts the binding of B56γ3 to MDM2, thereby inhibiting the ubiquitination degradation of B56γ3 and increasing the intracellular abundance of B56γ3, while strengths tumor-suppressive function of PP2A/AB56γ3C complex after DNA damage by promoting the interaction between PP2A-B56γ3 and p53, which results in p53 dephosphorylation at T55 and transcriptional activation; In breast cancer cells, metformin treatment can trigger the assembly PP2A-B56γ holoenzyme through phosphorylation of B56γ at S497,which leads to the inhibition of p70S6K-rpS6 axis to achieve metformin anti-cancer activity. In cardiomyocytes, β-adrenergic receptor stimulation induces phosphorylation of B56δ at S573 by PKA and increases the associated PP2A catalytic activity, possibly modulating the phosphorylation of specific PP2A-B56δ substrate. The absolute abundance of B56δ phosphorylation at S573 in mouse hearts with pressure overload-induced cardiac hypertrophy confirms the mechanistic role of B56δ in the pathogenesis of cardiac hypertrophy.

In addition to the variety of B56 subunits, the phosphorylation of B56 might contribute to the substrate specificity and biological functions of PP2A holoenzymes (Figure 1). It has been shown that phosphorylation of B56γ3 at Ser510 by ATM is an important modification that strengthens the tumor-suppressive function of the PP2A/AB56γ3C complex after DNA damage by promoting the interaction between PP2A-B56γ3 and substrate tumor suppressor P53, which results in P53 dephosphorylation at Thr55 and transcriptional activation [13]. Furthermore, phosphorylation of B56δ at Ser566 by PKA increases PP2A activity that catalyzes the dephosphorylation of DARPP-32 and thus coordinates the efficacy of dopaminergic neurotransmission in striatal neurons by the PP2A/AB56δC complex [15]. Phosphorylation of B56α at Ser41 by PKC plays a role in the pathophysiology of human heart failure through the inhibition of PP2A activity, resulting in IP3R1 activity and thus an increase in the IP3-mediated Ca^2+^ release from the endoplasmic reticulum [16]. In particular, the finding that some exogenous or endogenous substances exert their physiological effects by targeting B56 phosphorylation further emphasizes its importance, and more importantly, this provides a novel theoretical basis for the treatment of diseases associated with PP2A dysfunction. For example, it is proven that metformin may exert its anticancer activity through the phosphorylation of PPP2R5C at S497 that triggers PP2A activity and the subsequent dephosphorylation of p70S6K [17]. Similarly, β-adrenergic receptor (βAR)-induced phosphorylation of B56δ at S573 by PKA suggests its mechanistic role in the pathogenesis of cardiac hypertrophy, and the absolute abundance of B56δ phosphorylation at S573 in mouse hearts with pressure overload-induced cardiac hypertrophy reinforces this conclusion [18].

### 2.2. The Mechanism of B56 Phosphorylation Exerting Its Biological Effect

Given the potential (patho)physiological roles of B56 phosphorylation, understanding the molecular mechanism by which it functions is crucial for the development of drugs that target it. The phosphorylation of the B56 subunit functions by regulating the activity of PP2A, the localization of PP2A, and its binding to the target substrate. In terms of PP2A activity, it was found that B56 phosphorylation can either increase or decrease PP2A activity, depending on the distinct specific phosphosites of the regulatory subunit. For example, an increase in PP2A activity was observed for phosphorylation of B56δ at Ser566 or of B56γ at Ser497 and Ser510 [13,15,17]. On the contrary, phosphorylation of B56α at Ser41 or B56γ at Ser327 depresses PP2A activity [16,23]. The question of how the phosphorylation of B56 isoforms at multiple phosphoacceptor sites affects PP2A activity should be further explored. PP2A localization is also regulated by B56 subunit phosphorylation. For example, phosphorylation at Ser28 of B56α by PKR promotes mitochondrial localization of the PP2A-B56α complex [24]. Several studies have shown that B56 phosphorylation can affect either the expression of PP2A component subunits or its binding to A and C subunits. It has proven that phosphorylation of B56γ3 at Ser510 is necessary for the increase in abundance of B56γ3 after DNA damage [13]. Similarly, the phosphorylation of B56δ at Ser573 was associated with the expression of PP2A catalytic subunit C [18]. In addition, phosphorylated B56γ1 at Ser327 exhibited decreased binding to A and C subunits [23]. The mechanism may explain, at least in part, the effects of B56 subunit phosphorylation at different sites on PP2A activity.

In addition to regulating PP2A activity, another pathway through which B56 phosphorylation works is by altering its binding to the target substrate. It is well known that regulatory subunit B determines the substrate specificity and physiologic functions of the trimeric PP2A complex. In particular, the ability of B56 subunits to bind substrate is dictated by the phosphorylation of B56 subunits. It has been shown that phosphorylation of B56γ3 at Ser510 disrupts the binding of B56γ3 to MDM2 while promoting the interaction between B56γ3 and P53 [13]. However, so far, studies on B56 phosphorylation have mainly focused on the effect of phosphorylation on PP2A activity and the resulting modification in protein phosphorylation. The regulation of B56 phosphorylation on the phosphorylation status of specific PP2A-B56 substrates remains to be identified. There is an urgent need to understand the role of B56 phosphorylation in PP2A-B56 function, and once more mechanistic details are obtained, intervention in B56 phosphorylation might provide a new perspective for the treatment of diseases associated with PP2A-B56 dysfunction.

## 3. The Contribution of LxxIxE Motif to Decipher the Related Biological Functions of PP2A-B56

### 3.1. Overview of LxxIxE Motifs on the B56 Subunit Binding Protein

**Figure 2:**
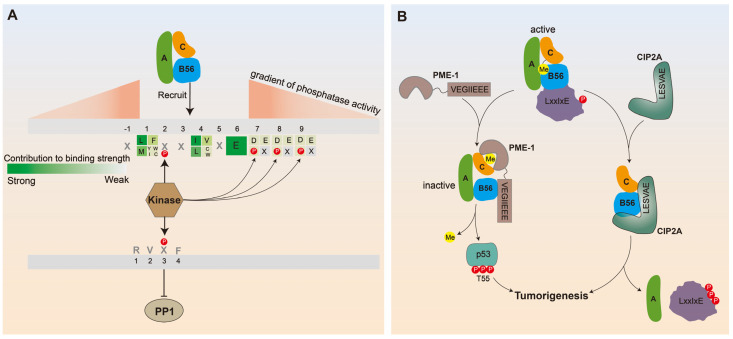
Biochemical function of PP2A-B56 interpreted by LxxIxE motif. (**A**) Prediction of PP2A-B56 targeting substrates and the binding strength: PP2A-B56 binds specifically to proteins containing LxxIxE motif, the strength of which depends on the composition of the amino acid at P1 and P4 ([LMFI]xx[ILV]xEx), and the presence of phosphorylated or acidic residues at P2, 7, 8 and 9. Prediction of PP2A-B56 phosphorylation site preference: the dephosphorylation ability of PP2A-B56 to different phosphorylation sites in the substrate depends on the distance of the LxxIxE motif relative to the phosphorylation site and its binding affinity to the substrate. In the figure, the shade of red is used to indicate the strength of phosphatase activity, and it can be seen that when the substrate motif composition is determined, the phosphorylation site closer to the motif exhibits a stronger ability to be dephosphorylated by PP2A-B56. Crosstalk among PP2A-B56, PP1, and kinase: PP2A-B56 binding to the LxxIxE motif can be enhanced by kinase phosphorylation, while PP1 interaction with the RVxF motif can be repressed by phosphorylation. Different responses to motif phosphorylation states make PP2A and PP1 associate with discrete regulators to guide different biological outcomes. (**B**) Mechanisms of CIP2A and PME-1 in PP2A inhibition and oncogenic functions: CIP2A hijacks B56 through its N-terminus, forming a disguised heterotrimer CIP2A-B56-PP2Ac, which not only causes the A subunit to be expelled but also shields B56 from binding to its substrate proteins with LxxIxE motif. PME-1 interacts with B56 through VEGIIEEE SLiM and triggers conformational changes in the PP2A holoenzyme. This will prompt the PP2Ac tail to move from the holoenzyme interface to the PME-1 active site and consequent holoenzyme demethylation and decommissioning.

Despite a myriad of structural and biochemical information available on PP2A holoenzymes, a scarcity of knowledge on substrate recognition by distinct PP2A holoenzymes due to the diverse regulatory subunits has been a critical barrier for the dissection of PP2A function in diverse cellular processes. The identification of conserved LxxIxE (where x represents any amino acid) motifs on the B56 subunit binding protein deciphers the mechanisms of PP2A substrate recognition and provides an important foundation for future efforts focused on interrogating the specific substrates and cellular function of distinct PP2A holoenzymes. LxxIxE, as the most common SLiM recruiting B56 subunits to their target protein, was revealed through a series of biochemical and structural studies, and variations were allowed at positions 1 and 4 ([LMFI]xx[ILV]xEx), but not in position 6. The binding affinity between B56 and the substrate can be modulated by the amino acid composition of P1 and P4 and by the presence of phosphorylated or acidic residues at P2, 7, 8, and 9. In detail, although variations are allowed at positions 1 and 4, there is a preference for leucine in P1 and isoleucine in P4. Moreover, at least one high-affinity determinant in either P1 or P4 and the invariant glutamic acid in P6 is necessary for B56 to bind to its substrate. Indeed, mutation of LxxIxE at P1 and P4 in RacGAP1 would disrupt its binding affinity for PP2A-B56 and, in turn, increase its phosphorylation and consequently cytokinesis [20]. Moreover, negatively charged residues D/E or S/T phosphorylation at positions 2, 7, 8, and 9 enhance the interaction. PP2A-B56 specifically binds the LxxIxE SLiM through a well-conserved hydrophobic pocket. In addition, there is an acidic surface near the LxxIxE pocket that is not hydrophobic but instead is highly negatively charged. Among the main regulatory substrates of PP2A-B56, a conserved basic charged rich region exists within ~15 amino acid N-terminal to an established LxxIxE motif, which is defined as a basic patch. When B56 binds to the LxxIxE motif through its hydrophobic pocket, the basic patch can also interact with the acidic surface to promote the binding of B56 to the substrate. Moreover, the binding contribution of the basic patch is independent of the strength of the LxxIxE motif. PP2A-B56 achieves substrate-specific recognition via the LxxIxE motif. For example, the pull-down experiment showed that a GST-CREB (99–159) fusion protein with LxxIxE motifs interacted with B56γ1, a member of the B56 family, rather than B55α, a member of the B55 family [25]. The study that the high-affinity LxxIxE motifs could only be co-purified with PP2A-B56 after cell expression and not others such as PP2A-B55α, PP2A-B55β or PP1γ further supports that LxxIxE motifs were specific for PP2A-B56 complexes [26]. The identification of the LxxIxE motifs not only expands the biological function of PP2A-B56 but also provides a reasonable explanation for the known biological phenomena related to PP2A-B56 (Figure 2).

### 3.2. Deciphering the Biological Function of PP2A-B56 through LxxIxE Motif

First of all, the discovery of the B56-binding motif LxxIxE not only predicts PP2A-B56 targeting proteins and/or substrates but also the substrates’ binding strength (Figure 2A). Using this SLiM motif, Wang et al. identified up to 100 potential PP2A-B56 targeting proteins and/or substrates [27]. Moreover, CIP2A, SENp6, and USP53 were predicted to be good binders for the B56 subunits, while GL12 was the weak binder based on the best consistent residues (L and I) at positions 1 and 4, and the quantity of negatively charged residues (D/E) at position 2, 7, 8, and 9 [28]. This prediction was also supported by the results of co-immunoprecipitation.

Secondly, the discovery of the B56-binding motif LxxIxE achieves the prediction of phosphorylation site preference of PP2A-B56 by using a specific PP2A-B56 inhibitor, namely 4× LxxIxE. These ultimately led to the conclusion that the distance of the LxxIxE motif relative to the phosphorylation site and its binding affinity to the substrate together determine the dephosphorylation ability of PP2A-B56 to different phosphorylation sites. Taking CDC20 as an example, by constructing CDC20 fragments containing a LxxIxE motif at variable distances (including 1× = 12 aa, 2× = 70 aa, and 4× = 130 aa) from Thr70 which is one of the phosphorylation sites located at CDC20, the researchers found that CDC20 4× displayed slower dephosphorylation kinetics compared to CDC20 1× and 2×. Furthermore, 1× GST-CDC20 fragment engineering to contain either a higher (KD = 1 μM) or lower (KD = 17 μM) affinity LxxIxE motif probe the importance of binding affinity, i.e., the higher affinity construct exhibited stronger dephosphorylation kinetics than the corresponding lower affinity construct [21]. Thus, these experiments favor a model where the PP2A-B56 binds to protein complexes containing LxxIxE motifs and a gradient of phosphatase activity is established around accessible phosphorylation sites depending on motif position and binding affinity (Figure 2A).

Third, it is well known that kinases and phosphatases maintain normal physiological function by coordinating the phosphorylation of proteins, the mechanism underlying this process, however, remains elusive. Increased recruitment of PP2A-B56 after the protein containing the LxxIxE motif is phosphorylated by the kinase provides a plausible explanation for this mechanism. In the case of Cyk4 (143-LSTIDESGS-151), which contains perfect consistent residues at positions 1 and 4, and one phosphorylation at position 7 by Plk1 appears to conceivably facilitate the interaction of Cyk4 with PP2A-B56 to direct the precise control of Cyk4 phosphorylation during cytokinesis [28]. Again, PP2A-B56 and PP1 are a major eukaryotic serine/threonine protein phosphatase involved in the regulation of numerous cell functions such as mitosis. It was found that PP2A-B56 and PP1 control different processes during kinetochore recruitment not due to their different localization, but opposite phospho-dependencies to phosphorylation inputs of their specific SLiMs. For example, the binding of PP1 to the RVxF motif (best-characterized SLiM for PP1) can be repressed by phosphorylation, whereas interaction of PP2A-B56 with the LxxIxE motif can be facilitated by phosphorylation (Figure 2A) [29]. Opposite phospho-dependencies allow PP2A-B56 and PP1 to participate in different biological processes at the kinetochore by regulating distinct substrates despite being recruited to quite similar molecular spaces. Thus, PP2A-B56 and PP1 respond in diametrically opposite ways to kinase-induced phosphorylation of their respective SLiM, making them functionally substitutes for each other and producing different phenotypic behaviors, which also facilitates the signaling integration of the kinase and phosphatase, and orchestrates sequential events for precise control of biological processes. 

Lastly, the identification of the B56-binding motif in PP2A modulators such as CIP2A and PME-1 reveals their mechanisms in PP2A inhibition and oncogenic functions (Figure 2B). It is suggested that CIP2A binds to PP2A-B56 (B56α and B56γ) through its N-terminus resulting in the expulsion of A-subunit and forming a disguised heterotrimer CIP2A-B56α-PP2Ac, thereby stabilizing CIP2A and promoting its tumorigenicity [30,31]. Excitingly, a motif that binds to B56 has been identified within the disordered region of CIP2A (N711RKLESVAEEHE725), although this motif exhibits a weaker affinity to B56 because the fourth position is V rather than I [28]. These results suggest a model where CIP2A not only hijacks B56 and C subunits to form heterotrimers but also mutes B56 in the CIP2A-B56α-PP2Ac trimer by shielding B56α from binding to its substrate proteins with LxxIxE motif. Moreover, the finding that the CIP2A peptide has a relatively low binding affinity to the B56 subunit also explains why CIP2A has anticancer activity only at high concentrations, where it can overcome the substrate binding to B56 [28,32,33]. Aside from CIP2A, PP2A methylesterase 1 (PME-1) has also been considered an endogenous PP2A inhibitor associated with various human cancers. It has been shown that substrate-mimicking SLiM (251VEGIIEE258E) of PME-1 plays a governing role in interaction with B56 and triggers PP2A holoenzyme conformational changes, causing PME-1 to come into dual contact with the PP2Ac active site and tail, and thus results in holoenzyme demethylation to prime holoenzyme for decommissioning [34]. Furthermore, the finding that mutations targeting the PME-1–B56 interface gave a similar or stronger effect than inhibitors of PME-1 on abolishing PME-1 activity and inducing a more rapid p53 accumulation accompanied by an attenuated increase in pThr55, suggested that the role of PME-1 in regulating pThr55 of p53 is mainly mediated by its interactions with PP2A-B56 holoenzymes [34]. These observations support the idea that p53 pThr55 is a target site of PP2A-B56 holoenzymes. Taken together, the identification of a B56-binding motif on CIP2A and PME-1 provides a coherent structural basis for their inhibitory to holoenzyme functions and new insights into mechanisms for their oncogenic function. 

## 4. Emerging Role of PP2A-B56 Complexes during Mitosis via the LxxIxE-Mediated B56 Binding

### 4.1. PP2A-B56 Is Recruited to the Strategic Place by Mitotic Regulators Containing Motifs to Achieve Mitotic Regulation

**Figure 3:**
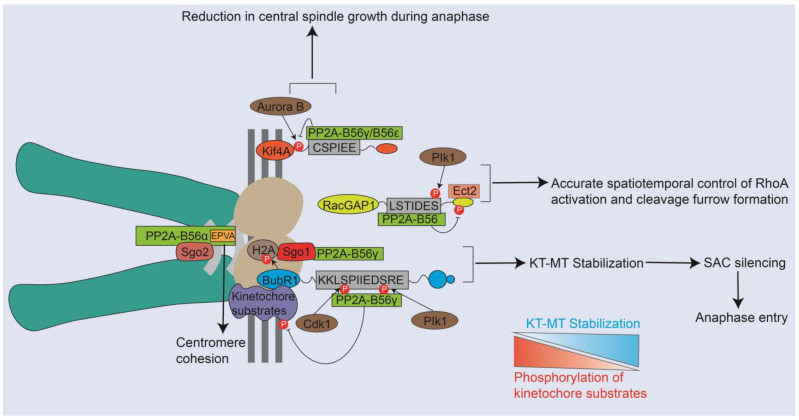
PP2A-B56 interacts with mitotic regulators via the LxxIxE motif to regulate several major mitotic processes. The phosphorylation of BubR1 by Cdk1 and Plk1 contributes to localizing PP2A-B56γ to the kinetochore, where kinetochore substrate phosphorylation is counteracted. Notably, the level of phosphorylation of kinetochore substrates is opposite to kinetochore-microtubule stabilization, which has been shown in gradated red and gradated blue, respectively, in the figure. Thus, recruitment of PP2A-B56γ to kinetochore results in decreased phosphorylation of kinetochore substrates and enhanced kinetochore-microtubule stabilization, which favors SAC silencing and anaphase entry. Sgo1 collaborates with BubR1 to maintain the presence of B56γ at the kinetochore, while B56α localizes primarily to the centromere to support proper centromeric cohesion via interaction with Sgo2 via a crucial EPVA signature. PP2A-B56γ/B56ε targets the central spindle through a Kif4A-dependent mechanism, counteracting the microtubule-stimulated ATPase activity of Kif4A by Aurora B and leading to a reduction in central spindle growth during anaphase. In response to Plk1-mediated multisite phosphorylation, RacGAP1 recruits PP2A-B56 for dephosphorylation and disrupts Ect2 binding, which may be critical for accurate spatiotemporal control of RhoA activation and cleavage furrow formation.

The successful segregation of chromosomes during mitosis is contingent upon the capacity of the spindle assembly checkpoint (SAC) to detect the attachment status of kinetochores and to postpone the initiation of anaphase until any inaccurately attached chromosomes have been rectified [35,36]. BubR1 plays a vital role in the accurate attachment of kinetochore-microtubules and the proper alignment of duplicated chromosomes during metaphase as a crucial constituent of SAC [37,38]. The crystal structure analysis of a complex formed by PP2A-B56 and BubR1 reveals that the KARD domain residues of BubR1 (667 KKLSPIIEDSREATH 681) possess consensus residues that perfectly align with the LxxIxE motif at positions 1, 4, and 7. Additionally, phosphorylation at position 2 (corresponding to BubR1 residues 670) and position 8 (corresponding to BubR1 residues 676) by Cdk1 and Plk1 significantly strengthen the interaction between BubR1 and B56γ [39,40]. This finding coincides with studies in which phosphorylation on the second or seventh positions of B56-specific SLiMs facilitates the interactions with B56 subunits [20]. The localization of PP2A-B56 to the kinetochore by BubR1 serves the purpose of counteracting substrates phosphorylation at the kinetochore, which is important for stabilization of kinetochore-microtubule attachments, and thereby SAC silencing and permitting anaphase entry [29,40,41]. Consistent with BubR1, KIF4A, and RacGAP1 have been shown to interact with PP2A-B56 via the LxxIxE motif as well, which contributes to the localization of B56 in mitosis, and the binding affinity is enhanced by mitotic phosphorylation [20,42]. Consequently, PP2A-B56γ and -ε employ a KIF4A-dependent mechanism to target the central spindle and rapidly dephosphorylate the KIF4A T799 via Aurora B kinase, thus counteracting microtubule-stimulated ATPase activity of KIF4A and resulting in a reduction in central spindle growth during anaphase [43]. Plk1-mediated multisite phosphorylation in Racgap1, a component of centralspindlin located in the spindle midzone, recruits both Ect2 (S157 and S164 at Racgap1) and PP2A-56 holoenzymes (S149 at Racgap1 corresponding to B56 binding motif P7). This recruitment subsequently leads to the dephosphorylation of Racgap1 and the disruption of Ect2 binding. This feedback mechanism might have an important role in accurate spatiotemporal control of RhoA activation and cleavage furrow formation [28]. Overall, mitotic regulators including BubR1, Kif4A, and RacGAP1 have been shown to interact with PP2A-B56 via the LxxIxE motif, which increases the concentration of this phosphatase at this strategic place and in return regulates several major mitotic processes (Figure 3). The structure of the PP2A B56-mitotic regulators complex provides vital insights into how the B56 subunit directs the recruitment of PP2A to specific substrates. 

### 4.2. The Participation of Different PP2A-B56 Complexes in Distinct Mitotic Events Was Decrypted from the Perspective of Motif

Noticeably, it has been demonstrated that different PP2A-B56 complexes (B56α, β, γ, δ, and ε) exhibit unique localization patterns and participate in distinct processes during mitosis through diverse isoform-specific interactions (Figure 3) [39,44]. The shugoshin (Sgo) proteins serve as universal protectors of centromeric cohesion and are recognized as scaffold proteins that interact with PP2A-B56 located in the kinetochore and centromere [42]. It has been reported that Sgo1 cooperates with BubR1 to sustain the presence of B56γ at the kinetochore and assists in preserving the Sgo2/B56α complex at the centromere. Specifically, BubR1 recruits B56γ to kinetochores through the LxxIxE motif, and it also initiates the phosphorylation of Histone-H2A to recruit Sgo1, which in turn favors anchoring B56γ at kinetochores for SAC silencing and chromosome alignment (Figure 3). Unlike B56γ, B56α localizes primarily to the centromere to support proper centromeric cohesion via a C-terminal stretch located between amino acids 405–453 containing a crucial EPVA signature that could promote the interaction with Sgo2 and the centromere and also fully inhibit binding to LxxIxE motifs and the kinetochore (Figure 3) [45]. These findings further support previous research indicating that B56γ and B56δ predominantly localize to kinetochores, while B56α, B56β, and B56ε are more likely to localize to centromeres [39]. Consequently, targeting specific PP2A-B56 isoforms holds promise as a feasible and valuable therapeutic approach. 

## 5. Motif-Based Understanding on Manipulation of PP2A-B56 by Diverse Viral Families

### 5.1. PP2A-B56 Is the Main PP2A Subfamily Attacked by Viruses

**Figure 4:**
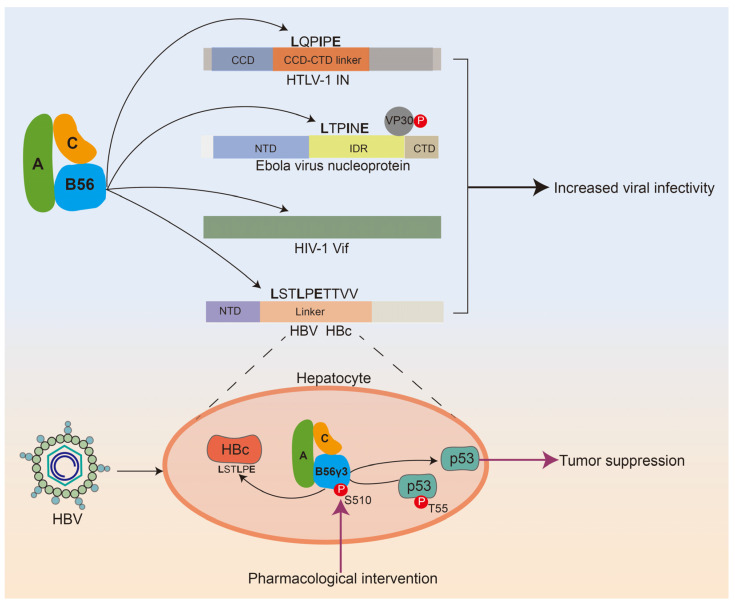
B56-binding motifs in various viruses make PP2A-B56 a target for viral attacks. HTLV-1 IN and HBV HBc recruit PP2A-B56 through the LxxIxE motif to increase viral infectivity. PP2A-B56 binds to the LxxIxE motif of Ebola virus nucleoprotein, which serves as a scaffold to recruit VP30 for dephosphorylation, causing viral transcription. The recognition and interaction of HIV-1 Vif by PP2A-B56 involve residues surrounding the LxxIxE SLiM binding pocket, and the resulting degradation of B56 is beneficial to HIV-1 pathogenesis. In HBV-infected hepatocytes, phosphorylation of S510 targeting B56γ3 increases the abundance of B56γ3-specific PP2A complex and enhances the interaction of the PP2A-B56γ3 complex with p53, leading to T55 dephosphorylation and p53 activation. Thus, S510 of B56γ3 can be considered as a target for pharmacological intervention against HBV-induced HCC.

Many viruses manipulate the activity of PP2A and downstream signaling pathways to their advantage by binding to various component subunits of PP2A, and therefore PP2A is considered a virus-host factor [46,47,48]. The classic example is that the small T antigen (ST) of simian virus 40 (SV40) interacts with the PP2A scaffolding A by displacing regulatory B subunits, thereby inhibiting PP2A-mediated dephosphorylation of numerous substrates and ultimately promoting viral replication [49]. In particular, the great majority of viral proteins believed to engage with the regulatory subunit of PP2A exclusively target B56 [47,48,50]. For instance, Co-IP analysis verified the specific interaction between B56δ and HCV NS5B, which is essential for HCV infection in hepatoma cells by promoting HCV replication [50]. In recent years, the identification of B56 binding motifs LxxIxE in various viruses has provided a reasonable explanation for this phenomenon and is also conducive to understanding how viral hijacking of PP2A-B56 can produce different biochemical and phenotypic events (Figure 4).

### 5.2. The Discovery of Binding Motifs in Various Clinically-Relevant Viruses Revealed Why PP2A-B56 Was Hijacked by the Virus

The Hepatitis B virus (HBV) is responsible for causing various liver-related conditions such as viral hepatitis, further liver fibrosis, cirrhosis, and even malignancy [51,52,53,54]. Within the viral capsid protein (HBc), there exists an N-terminal domain (NTD) and a C-terminal domain (CTD), which are connected by a short linker peptide. This linker peptide contains a consensus binding motif (139 LSTLPETTVV 149) that facilitates the interaction with PP2A-B56. It has been documented that the HBc linker peptide exerts its essential and multiple functions partially through the recruitment of PP2A-B56 to modulate HBc phosphorylation and multiple stages of HBV replication [55]. An additional illustration of viral B56 recruitment through the LxxIxE SLiM was observed in the Human T-cell lymphotropic virus type 1 (HTLV-1). HTLV-1 is a deltaretrovirus that is one of the most oncogenic human viruses in existence [56]. Integrase (IN) is responsible for facilitating the integration of viral genetic material into the host genome, a crucial step in the replication cycle of deltaretroviruses. The region connecting the IN catalytic domain (CCD) and the C-terminal domain (CTD) contains the LxxIxE sequence. It has been reported that B56γ is recruited to the delta retroviral intasome via the LxxIxE SLiM in the IN/CCD-CTD linker and is therefore considered an important host factor for the virus [48]. Indeed, the infectivity of HTLV-1 was markedly reduced by shRNA knockdown of B56γ [57]. Although the well-known function of HIV-1 Vif is to degrade antiviral APOBEC3 enzymes, which are considered the most ancient and antagonistic substrate of Vif, recent research on HIV-1-infected T cells revealed new Vif substrates, including several members from the B56 family [58,59]. These studies have also demonstrated that Vif initiates G2/M arrest by degrading multiple B56 [60,61]. Notably, the degradation of B56α by Vif occurs at a rate comparable to that of APOBEC3G, and this activity is prevalent in Vif isolates derived from patients. These findings further emphasize the significance of B56 degradation in the pathogenesis of HIV-1. Intriguingly, despite the absence of a LxxIxE SLiM motif in Vif, the co-expression of a LxxIxE-like peptide inhibitor, which has been demonstrated to outcompete B56 binders, resulted in a dose-dependent inhibition of Vif-mediated degradation of B56α. However, this inhibition was not observed in the case of APOBEC3G, indicating that the LxxIxE-like peptide is specific to the Vif/B56 interaction [60]. Furthermore, the recognition of Vif by B56 appears to involve residues surrounding the LxxIxE SLiM binding pocket. Previous studies have shown that proteins containing LxxIxE motifs can serve as scaffolds for recruiting other proteins for dephosphorylation purposes. For example, the Ebola virus (EBOV), which is considered the most clinically significant filovirus, relies on the unphosphorylated form of the viral transcription factor VP30 for its transcription. Kruse et al. have demonstrated that the Ebola virus nucleoprotein (NP) contains an LxxIxE motif that interacts with PP2A-B56, serving as a scaffold for the recruitment of VP30 for dephosphorylation and subsequently facilitating viral transcription [26]. In fact, it has been observed that targeting the LxxIxE-binding pocket of PP2A-B56 effectively inhibits Ebola virus transcription and proliferation. In summary, the discovery of distinct binding motifs in many clinically relevant viruses for PP2A-B56 as well as the role of LxxIxE-mediated B56 binding in virus infection extend our insights into viral PP2A targeting, which will suggest generating specific PP2A-B56 inhibitors might be an effective antiviral strategy.

### 5.3. B56γ Phosphorylation at Ser510 Is Predicted to be an Effective Intervention against HBV-Induced HCC

Hepatocellular carcinoma (HCC) is one of the most common malignancies and is among the top three contributors to malignant mortality [62]. Chronic HBV infection is responsible for a significant proportion (50–80%) of HCC cases [62]. As previously stated, the PP2A regulatory subunit B56 serves as a functional binding partner for various clinically relevant viruses, including HBV, HTLV-1, HIV-1, and the Ebola virus, through the B56-binding LxxIxE SLiM (Figure 4). This interaction plays a crucial role in virus infection, indicating that B56 holds potential as a biomarker and target for antiviral pharmacological interventions. In the B56 family, the expression of B56γ exhibited a positive correlation with HBx levels in HBV-infected human-liver-chimeric mice or cells and HBx-expressing mice or hepatic cells [63]. In the clinical samples analyzed, the expression levels of HBx and B56γ were found to be significantly lower in HBV-associated HCC tumor tissues compared to peritumor tissues. Notably, the suppression of B56γ expression was observed to enhance xenograft tumor growth and migration of HBx-expressing HCC cells in mice models. Conversely, the genetic upregulation of B56γ demonstrated inhibitory effects on cell growth, migration, and invasion in HBx-expressing HCC cells [64]. The mechanistic evidence demonstrates that B56γ facilitates cell cycle arrest in a p53/p21 pathway-dependent manner, leading to apoptosis in HBx-expressing HCC cells. This effect is achieved by dephosphorylating p-p53 Thr55, thereby promoting p53 activation. These suggested the functional importance of B56γ in HBV-related hepatic injury via regulating the activity of tumor suppressor P53 (Figure 4) [63,64]. Of note, we have mentioned earlier that Ser510 phosphorylation promotes the anti-tumor effects of PP2A-B56γ through increasing the abundance of B56γ3 and facilitating the interaction between PP2A-B56γ3 complex and its substrate, p53 (Figure 1) [13]. Thus, these findings strongly suggested modification of the phosphorylation of B56γ at the S510 site might present an opportunity for intervention in viral, especially HBV-induced HCC.

## 6. The Contribution of PP2A-B56 to Tumor Suppression and its Dual Role in Breast Cancer through LxxIxE Motif Mimicry

### 6.1. The PP2A-B56 Subfamily Plays an Important Role in Various Tumor Inhibition

The tumor suppressor function of PP2A has been extensively documented in various studies [65,66,67]. In vivo evidence supporting this notion is provided by the PPP2R5D knockout mice, which lack the expression of the PP2A B56δ subunit and exhibit a predisposition to spontaneous hematologic malignancy and HCC [68]. In lung cancer, the F395C mutation was identified to disrupt the B56γ-p53 interaction, leading to the failure in the p53-dependent tumor-suppressive function of PP2A by inhibiting p53 Thr55 dephosphorylation and p21 transcription [69]. A reduction in the expression of PPP2R5A and PPP2R5C, the genes responsible for encoding B56α and B56γ, respectively, was observed in metastatic tissues of melanoma, resulting in the overexpression of C-MYC occurred and the suppression of oncogene-induced senescence [70,71]. It is well-known that the methylation of PP2A Leu309 enhances the association of regulatory B subunits with the PP2A core enzyme [9]. Loss of LCMT1 was reported to facilitate prostate cancer progression and therapy resistance by decreasing methyl-PP2A-C levels, affecting the binding affinity of B56α and activating subsequent androgen receptor signaling [72]. Aside from site deletion mutations and epigenetic regulation, PP2A can also be functionally impaired by the upregulation of exogenous and endogenous inhibitors in cancer [65]. The small t antigen (ST) of the DNA tumor virus SV40 acts as an exogenous regulator and promotes cell proliferation through the disruption of PP2A phosphatase activity, which occurs mainly by binding to the PP2A core enzyme and a lesser degree by displacing the B56γ subunit from the holoenzyme, perturbing its substrate selectivity [73]. In cancers, the best characterized endogenous inhibitors of PP2A are SET and CIP2A. The SET was reported to inhibit a specific PP2A holoenzyme composed of PP2A-Aβ, PP2A-B56γ, and PP2A-Cα in A549 cells [74]. The SET-PP2AC stability can be enhanced by the dephosphorylation of SET Ser171 [75]. As already indicated, the dimerization of CIP2A and its N-terminal region contribute to the maximal interaction with PP2A tumor suppressor subunits B56α and B56γ [30]. As a long-lived protein, CIP2A is a potential therapeutic target among 12 different cancers [76]. In breast cancer, the lncRNA-encoded micropeptide CIP2A-BP exhibited a direct binding affinity towards CIP2A, thereby the inhibition of PI3K-AKT-NFκB pathway led to a decrease in MMP2, MMP9, and Snail expression [77]. Of note, the studies above highlighted the importance of the B56 subunit. Likewise, the specific combination of SET with B56α, but not with B55α and PR72 works in E2F1 transcription in gastric cancer [78]. Among 11 B subunits, only B55α, B56α, and B56ε are involved in ST-induced cell malignant transformation [79]. Accordingly, targeting the PP2A-B56 family is an exciting research field with promising potential for innovative cancer therapy.

### 6.2. Motif-Based Decryption of PP2A-B56 Promoting DNA Damage Repair and Migration in Breast Cancer

**Figure 5:**
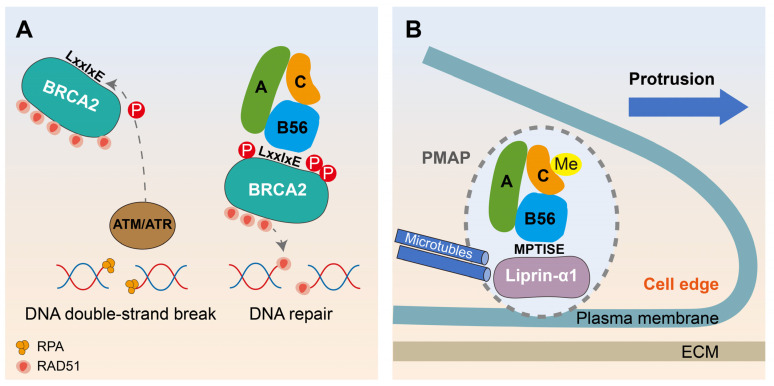
The dual role of PP2A-B56 in breast cancer. (**A**) In response to DNA damage, single-stranded DNA (ssDNA) is rapidly surrounded by replication protein A (RPA), forming RPA-ssDNA filaments. The phosphorylation of BRCA2 by DNA damage kinases (ATM/ATR) facilitates the binding of PP2A-B56 via the LxxIxE motif, thus triggering recruitment of the BRCA2-PP2A-B56 complex to broken DNA. BRCA2 acts by controlling RAD51 filament formation on ssDNA-RAD51 filaments, facilitating the DNA damage repair process in breast cancer. (**B**) The C-terminal was virtually completely methylated to maintain the PP2A holoenzyme assembly in the highly metastatic breast cancer cells. The recruitment of PP2A-B56γ at plasma membrane-associated platform (PMAP) near the edge of cancer cells induced by scaffold protein liprin-α1 through LxxIxE motif supports the protrusion and invasion of breast cancer cells on extracellular matrix (ECM).

Interestingly, there exists a dual role of PP2A-B56 in breast cancer (Figure 5). On the one hand, the tumor-suppressing role of BRCA2 in breast cancer relies on the proper spatiotemporal formation of the BRCA2/PP2A-B56 complex. Specifically, in response to DNA damage, single-stranded DNA (ssDNA) is rapidly surrounded by replication protein A (RPA), forming RPA-ssDNA filaments. The phosphorylation of BRCA2 on Ser1106, Ser1123, and Thr1128 by DNA damage kinases ATM and ATR facilitates the binding of PP2A-B56 via LxxIxE motifs. The phosphorylation triggers recruitment of the BRCA2/PP2A-B56 complex to broken DNA which is required for the efficient formation of RAD51 nucleoprotein filament on fractured ssDNA and homologous recombination-mediated repair of toxic DNA double-strand breaks [80] (Figure 5A). On the other hand, the interaction between liprin-α1 and B56γ regulatory subunit supports breast cancer cell motility. Scaffold liprin-α1 is required to assemble the dynamic plasma membrane-associated platform (PMAP) [81], which represents a means to dynamically localize protein scaffolds and enzymes to regulate events at the front of motile cells. Liprin-α1 interacts via the N-terminal short linear motif (6 MPTISE 11) with the PP2A-B56γ in migrating breast cancer cells. Silencing of either B56γ or liprin-α1 results in a similar weakening effect on lamellipodia dynamics. Interestingly, PP2A-C was virtually completely methylated in the highly metastatic MDA-MB-231 breast cancer cells to maintain the formation of stable B56γ/PP2A-C/PP2A-A complexes. Thus, the enrichment of PP2A-B56γ induced by liprin-α1 at the PMAP maintains the heterotrimer of PP2A holoenzyme and enables the protrusive activity of invasive tumor cells [82] (Figure 5B). Overall, more prospective basic studies and clinical trials are needed to place PP2A-B56 in a reasonable position for anti-cancer effects.

## 7. Conclusions

PP2A is an essential protein phosphatase and participates in various cellular processes via the formation of ~100 heterotrimeric holoenzymes containing distinct substrate-determining regulatory ‘‘B’’ subunits. However, the lack of knowledge about substrate recognition by multiple PP2A holoenzymes has considerably hindered the dissection of PP2A function and thus the treatment of diseases associated with PP2A dysfunction. Indeed, small molecule drugs that selectively bind and stabilize a single B-subunit containing heterotrimer, B56α, have enormous clinical potential as a therapeutic strategy due to their ability to drive dephosphorylation of select pathogenic substrates [83,84]. Therefore, discriminating the contributions of PP2A composed of different regulatory subunits in cells is key to deciphering PP2A (patho)physiology and, particularly, the therapeutic potential of PP2A targeting in disease. This review mainly focuses on PP2A-B56 because of the recent discovery of a LxxIxE motif that achieves PP2A-B56 specific binding, and the phosphorylation properties of B56 that regulate its ability to bind substrates. In summary, this review illustrates the following conclusions:

1. B56 phosphorylation plays an important role in a variety of biological processes by regulating PP2A activity and its ability to bind substrates.
2. The discovery of a LxxIxE motif that achieves PP2A-B56 specific binding and deciphers the substrate and phosphorylation site preference regulated by PP2A-B56, and the interaction mechanism between phosphatases and kinases.
3. The discovery of B56-binding motifs in mitotic regulators not only decrypts the role of PP2A-B56 in mitosis, but more importantly, sheds light on the reasons why different PP2A-B56 complexes participate in different mitotic events.
4. LxxIxE-mediated B56 binding in virus infection makes PP2A-B56 a key target for virus manipulation, and the elevation of B56γ phosphorylation at S510 is predicted to be an effective treatment for HBV-induced HCC.
5. LxxIxE motif-based decryption of PP2A-B56 promoting DNA damage repair and migration expands the understanding of the PP2A-B56 function on cancer.

## 8. Future Perspectives

Although it has been established that PP2A-B56 is recruited to its strategic place and performs corresponding biological functions through LxxIxE motif-mediated binding, the functionally relevant substrates regulated by motif-bound PP2A-B56 have not been identified. For example, recruitment of PP2A-B56γ at PMAPs by liprin-α1 has been shown to regulate tumor cell migration, but the functional substrate(s) of liprin-α1-bound PP2A-B56γ on cell motility have not been found [82]. Similarly, the functionally relevant substrates regulated by BRCA2-bound PP2A-B56 in DNA repair or by BubR1-bound PP2A-B56 in mitosis have not been able to be identified [80]. Future work will aim at determining how the recruitment of PP2A-B56 in a LxxIxE motifs-dependent manner exerts their action towards disease-specific substrates.

As the primary source of serine/threonine phosphatase activity in eukaryotic cells, PP2A plays a key role in many biological processes including phosphorylation signaling cascades. Based on the extensive role of PP2A, its dysregulation undoubtedly contributes to disease, which has made PP2A an attractive target in disease treatment, especially cancer treatment, in the past decade. A prime example is the endogenous PP2A inhibitors SET, which binds to and inactivate the catalytic subunit C of PP2A and is often overexpressed in cancer. Several molecules targeting this negative regulator, such as FTY720, CM-1231and OP449, display anti-cancer activity by causing the intracellular release of SET from PP2A and consequently, indirectly reactivating PP2A [83,85]. However, the therapeutic strategy for indiscriminate PP2A activation has been limited by the structural diversity of the PP2A family. Therefore, there is a need for a more complete understanding of the role of each PP2A complex in cancer, as well as the high-resolution structure of each heterotrimer, in order to selectively target specific holoenzymes against disease-specific substrates. It was found that DT-061, a PP2A activator that specifically stabilizes the PP2A-B56α holoenzyme and that induces cell death and attenuates tumor growth through dephosphorylating selective substrates [84,86,87]. The specificity of DT-061 to a single member of the B56 family illustrates that small molecules could allow for the specific reestablishment of PP2A homeostasis for cancer treatment. In addition, the combination of DT-061, FTY720, and CM-1231 plus venetoclax, an FDA-approved anti-leukemia drug, has synergistic antileukemic responses by reducing the expression of p-Bcl2, p-ERK, and MCL-1 [88]. Knocking out B56α rather than B55α in HL-60 cells by CRISPR-Cas9 assays abolished the synergistic effects, suggesting that the PP2A-B56α complex is the main regulator of this synergistic effect [88]. Together, these new strategies for activating PP2A will aid in the development of PP2A-specific drugs for cancer treatment.

## Data Availability

Not applicable.

## Abbreviations

βAR: β-adrenergic receptor; CCD, catalytic domain; CIP2A, cancerous inhibitor of protein phosphatase 2A; CTD, C-terminal domain; EBOV, Ebola virus; HBV, Hepatitis B virus; HBc, viral capsid protein; HCC, Hepatocellular carcinoma; HTLV-1, Human T-cell lymphotropic virus type 1; IN, Integrase; NTD, N-terminal domain; NP, nucleoprotein; SLiM, specific short linear motif; PP1, protein serine/threonine phosphatase 1; PP2A, protein serine/threonine phosphatase 2A; PME-1, PP2A methylesterase 1; RPA, replication protein A; SAC, spindle assembly checkpoint; Sgo, shugoshin; ST, small T antigen; SV40, simian virus 40.
